# 1-(3-*p*-Tolyl­isoxazol-5-yl)cyclo­hexa­nol

**DOI:** 10.1107/S1600536809044900

**Published:** 2009-11-04

**Authors:** Ouafaa Khalil, Khalid Bougrin, Rachid Benhida, Mohamed Soufiaoui, Lahcen El Ammari

**Affiliations:** a Laboratoire de Chimie des Plantes et de Synthèse Organique et Bioorganique, Département de Chimie, Faculté des Sciences BP, 1014 Rabat, Morocco; b Laboratoire de Chimie des Molécules Bioactives et des Arômes, UMR-CNRS 6001, Institut de Chimie de Nice, Université de Nice-Sophia Antipolis, Parc Valrose, F-06108 Nice Cédex 2, France; c Laboratoire de Chimie du Solide Appliquée, Faculté des Sciences, Université Mohammed V-Agdal, 4, Avenue Ibn Battouta, BP 1014, Rabat, Morocco

## Abstract

The title compound, C_16_H_19_NO_2_, contains two mol­ecules in the asymmetric unit. Each mol­ecule is composed of three inter­connected rings, two essentially planar rings, *viz.* the isoxazole and the methyl­benzyl aromatic ring [maximum deviations of 0.0027 (13) and 0.0031 (19) Å from the isoxazole and methylbenzyl ring planes, respectively, in the first molecule, 0.0018 (12) and 0.019 (2) Å in the second molecule], and one cyclo­hexa­nol ring having a chair conformation. Although the two mol­ecules have similar bond distances and angles, they differ in the orientation of the cyclo­hexa­nol ring with respect to the tolyl­isoxazole unit. In the first mol­ecule, the dihedral angle between the isoxazole and methyl­benzyl rings is 22.03 (8)° and between the isoxazole and cyclo­hexa­nol rings is 30.15 (8)°. The corresponding values in the second mol­ecule are 6.13 (10) and 88.44 (8)°, respectively. In the crystal, the molecules are linked by O—H⋯O and O—H⋯N hydrogen bonds, building up a zigzag chain parallel to the *a* axis.

## Related literature

For isoxazole derivatives as building blocks in organic synthesis and combinatorial chemistry, see: Tu *et al.* (2009 ▶); Tang *et al.* (2009 ▶). For their biological activity, see: Deng *et al.* (2009 ▶); Kozikowski *et al.* (2008 ▶); Lee *et al.* (2009 ▶).
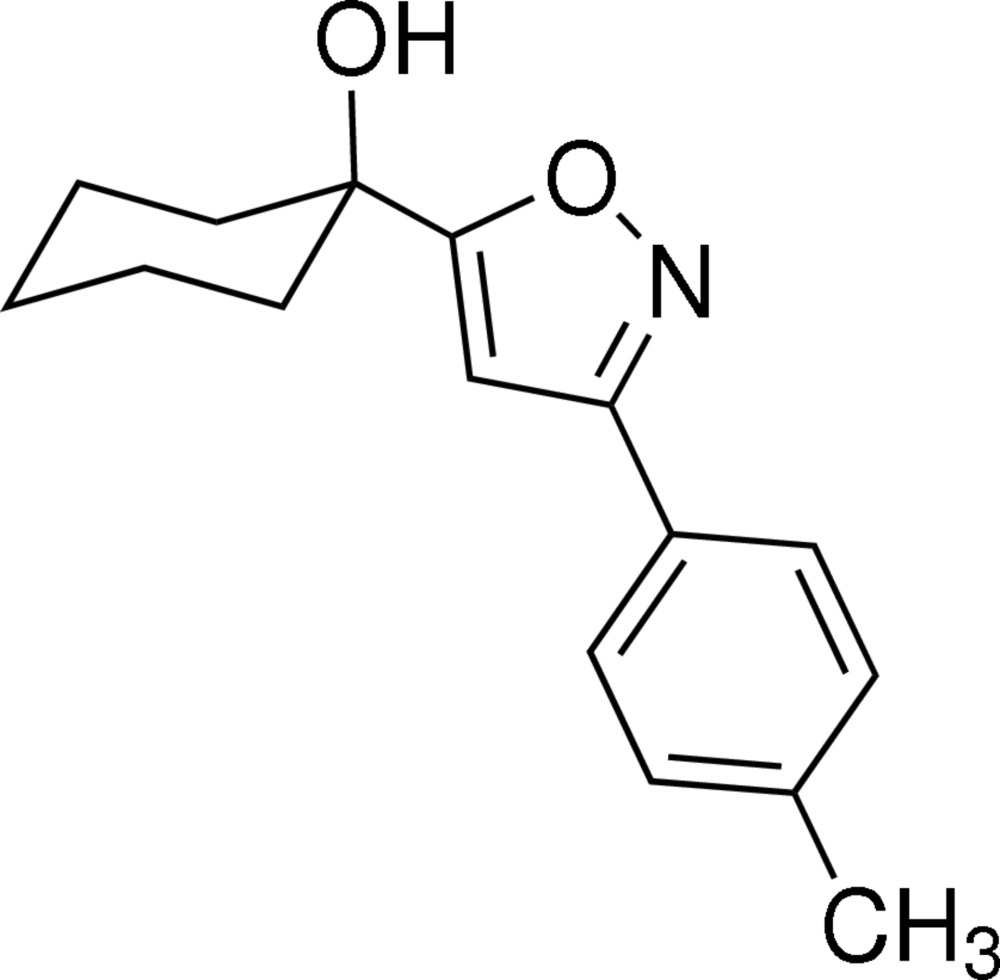


## Experimental

### 

#### Crystal data

C_16_H_19_NO_2_
*M* *_r_* = 257.32
Orthorhombic, 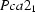

*a* = 10.9404 (3) Å
*b* = 9.7136 (3) Å
*c* = 26.9207 (7) Å
*V* = 2860.88 (14) Å^3^
*Z* = 8
Mo *K*α radiation
μ = 0.08 mm^−1^
*T* = 298 K
0.18 × 0.17 × 0.10 mm

#### Data collection

Bruker X8 APEXII diffractometer
Absorption correction: none
87116 measured reflections
4377 independent reflections
3820 reflections with *I* > 2σ(*I*)
*R*_int_ = 0.032

#### Refinement

*R*[*F* ^2^ > 2σ(*F* ^2^)] = 0.039
*wR*(*F* ^2^) = 0.105
*S* = 1.04
4377 reflections
347 parameters
1 restraint
H-atom parameters constrained
Δρ_max_ = 0.20 e Å^−3^
Δρ_min_ = −0.15 e Å^−3^

### 

Data collection: *APEX2* (Bruker, 2005 ▶); cell refinement: *SAINT* (Bruker, 2005 ▶); data reduction: *SAINT*; program(s) used to solve structure: *SHELXS97* (Sheldrick, 2008 ▶); program(s) used to refine structure: *SHELXS97* (Sheldrick, 2008 ▶); molecular graphics: *ORTEP-3 for Windows* (Farrugia, 1997 ▶) and *PLATON* (Spek, 2009 ▶); software used to prepare material for publication: *WinGX* (Farrugia, 1999 ▶).

## Supplementary Material

Crystal structure: contains datablocks I, global. DOI: 10.1107/S1600536809044900/dn2504sup1.cif

Structure factors: contains datablocks I. DOI: 10.1107/S1600536809044900/dn2504Isup2.hkl

Additional supplementary materials:  crystallographic information; 3D view; checkCIF report

## Figures and Tables

**Table 1:** Hydrogen-bond geometry (Å, °)

*D*—H⋯*A*	*D*—H	H⋯*A*	*D*⋯*A*	*D*—H⋯*A*
O1—H1⋯O2	0.82	1.98	2.7892 (12)	168
O2—H2⋯N2^i^	0.82	2.05	2.8629 (16)	173

Symmetry code: (i) 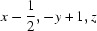.

**Fig. 1.**
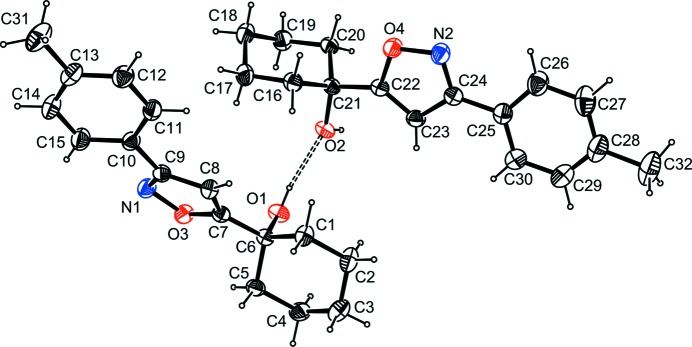
: Molecular view of the asymmetric unit with the atom labeling scheme. Displacement ellipsoids are drawn at the 30% probability level. H atoms are represented as small spheres of arbitrary radii. Hydrogen bond is shown as dashed line.

**Fig. 2.**
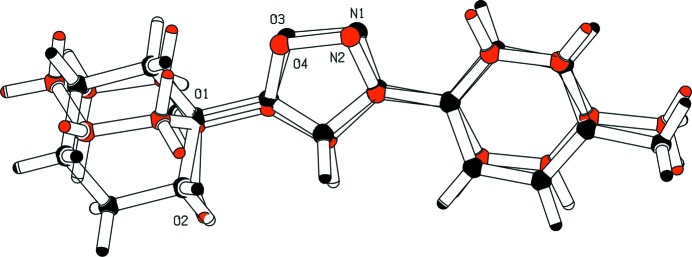
: View showing the fitting of the two molecules building the asymmetric unit.

**Fig. 3.**
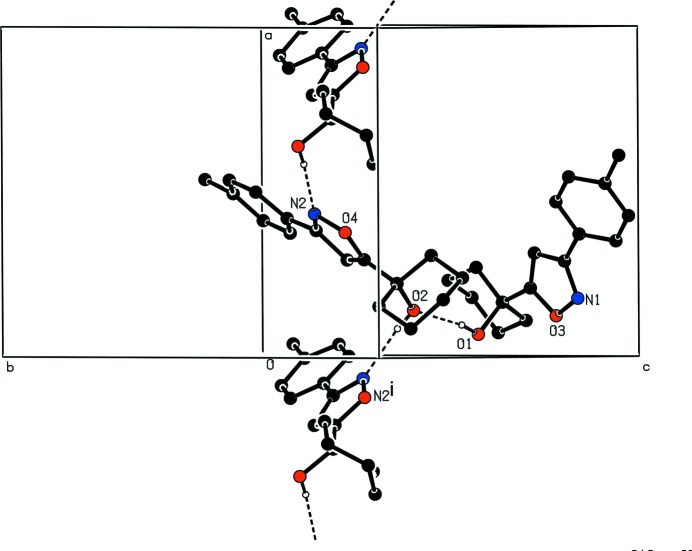
: Partial packing view showing the formation of a chain through O—H···N hydrogen bonds shown as dashed lines. H atoms not involved in hydrogen bondings have been omitted for clarity. [Symmetry code: (i) -x-1/2, y+1, z+1/2]

## supplementary crystallographic information

### Comment

Isoxazole derivatives are important class of heterocyclic compounds and their chemical and biochemical properties have been extensively studied. They have served as a versatile building blocks in organic synthesis and combinatorial chemistry (Tu *et al.* 2009, Tang *et al.* 2009). Isoxazole systems have also been targeted in synthetic investigations for their known biological and pharmacological properties such as hypoglycemic, anti-inflammatory and anti-bacterial activities. Recently, the growing interest in such analogues also rises from their high potential value as antiviral (Deng *et al.* 2009, Lee *et al.* 2009) and anti-tumor agents (Kozikowski *et al.* 2008).

We have undertaken the X-ray diffraction study of the title compound, in order to understand the molecular features which stabilize its observed conformation. The asymmetric unit contains two molecules crystallographically independent. Each molecule is formed by three interconnected cycles, two essentially planar rings: isoxazole and methylbenzyl rings while the 3rd ring (cyclohexanol) has a chair conformation (Fig. 1). The difference between the molecules lies in the orientation of the rings in each molecule as shown in the fitting drawing (Fig. 2) obtained with PLATON (Spek, 2003). Thus in the first molecule (C1 to C15) the dihedral angles between the isoxazole ring and methylbenzyl ring planes is 22.03 (8)° and between the isoxazole and cyclohexanol ring planes is 30.15 (8)°. Whereas in the second molecule (C16 to C30), equivalent angles have as values 6.13 (10) and 88.44 (8)°, respectively.

The two molecules within the asymmetric unit are linked through O-H···O hydrogen bond building a pseudo dimer. These pseudo dimers are further linked to each other by O-H···N hydrogen bonds forming a zig-zag like chain parallel to the a axis (Table 1, Fig. 3).

### Experimental

A mixture of 1-ethynylcyclohexanol (1 mmol) and *p*-methylbenzylaldoxime (1.2 mmol) was dissolved in CH_2_Cl_2_ (20 ml), the solution was then cooled thoroughly with ice at 0–5°C. 15 ml of sodium hydroxide solution (12 g. of sodium hydroxide per 100 g. of water) were gradually added under vigorously stirring for 5 h. The organic phase was separated and dried over anhydrous sodium sulfate, filtered and the solvent evaporated under reduced pressure. The residue was purified by recrystallization from ethanol. The structure of adduct was confirmed by spectroscopic methods.

### Refinement

All H atoms attached to C atoms and O atom were fixed geometrically and treated as riding with C—H = 0.93Å (aromatic), 0.96 Å (methyl) or 0.97 Å (methylene) and O—H = 0.82 Å with U_iso_(H) = 1.2U_eq_(aromatic, methylene) or U_iso_(H) = 1.5U_eq_(methyl,O).

In the absence of significant anomalous scattering, the absolute structure could not be reliably determined and then the Friedel pairs were merged and any references to the Flack parameter were removed.

### Crystal data

**Table:**

C_16_H_19_NO_2_	*F*(000) = 1104
*M**_r_* = 257.32	*D*_x_ = 1.195 Mg m^−^^3^
Orthorhombic, *P**c**a*2_1_	Mo *K*α radiation, λ = 0.71073 Å
Hall symbol: P 2c -2ac	Cell parameters from 4377 reflections
*a* = 10.9404 (3) Å	θ = 2.6–30.3°
*b* = 9.7136 (3) Å	µ = 0.08 mm^−^^1^
*c* = 26.9207 (7) Å	*T* = 298 K
*V* = 2860.88 (14) Å^3^	Bloc, colourless
*Z* = 8	0.18 × 0.17 × 0.10 mm

### Data collection

**Table:**

Bruker X8 APEXII diffractometer	3820 reflections with *I* > 2σ(*I*)
Radiation source: fine-focus sealed tube	*R*_int_ = 0.032
graphite	θ_max_ = 30.3°, θ_min_ = 0.8°
φ and ω scans	*h* = −15→15
87116 measured reflections	*k* = −13→13
4377 independent reflections	*l* = −38→38

### Refinement

**Table:**

Refinement on *F*^2^	Primary atom site location: structure-invariant direct methods
Least-squares matrix: full	Secondary atom site location: difference Fourier map
*R*[*F*^2^ > 2σ(*F*^2^)] = 0.039	Hydrogen site location: inferred from neighbouring sites
*wR*(*F*^2^) = 0.105	H-atom parameters constrained
*S* = 1.04	*w* = 1/[σ^2^(*F*_o_^2^) + (0.0535*P*)^2^ + 0.2498*P*] where *P* = (*F*_o_^2^ + 2*F*_c_^2^)/3
8578 reflections	(Δ/σ)_max_ = 0.008
347 parameters	Δρ_max_ = 0.20 e Å^−^^3^
1 restraint	Δρ_min_ = −0.15 e Å^−^^3^

### Special details

**Table:**

Geometry. All esds (except the esd in the dihedral angle between two l.s. planes) are estimated using the full covariance matrix. The cell esds are taken into account individually in the estimation of esds in distances, angles and torsion angles; correlations between esds in cell parameters are only used when they are defined by crystal symmetry. An approximate (isotropic) treatment of cell esds is used for estimating esds involving l.s. planes.
Refinement. Refinement of *F*^2^ against ALL reflections. The weighted *R*-factor *wR* and goodness of fit *S* are based on *F*^2^, conventional *R*-factors *R* are based on *F*, with *F* set to zero for negative *F*^2^. The threshold expression of *F*^2^ > σ(*F*^2^) is used only for calculating *R*-factors(gt) *etc*. and is not relevant to the choice of reflections for refinement. *R*-factors based on *F*^2^ are statistically about twice as large as those based on *F*, and *R*- factors based on ALL data will be even larger.

### Fractional atomic coordinates and isotropic or equivalent isotropic displacement parameters (Å^2^)

**Table:**

	*x*	*y*	*z*	*U*_iso_*/*U*_eq_	
O1	0.06445 (8)	0.00823 (10)	0.57893 (4)	0.0480 (2)	
H1	0.0930	0.0832	0.5867	0.072*	
O3	0.11978 (9)	−0.17692 (11)	0.65818 (4)	0.0483 (2)	
N1	0.17299 (11)	−0.19192 (14)	0.70537 (4)	0.0503 (3)	
C1	0.26673 (12)	−0.03462 (16)	0.54425 (5)	0.0477 (3)	
H1A	0.3325	−0.1015	0.5438	0.057*	
H1B	0.2974	0.0489	0.5595	0.057*	
C2	0.22715 (16)	−0.0037 (2)	0.49105 (6)	0.0619 (4)	
H2A	0.1661	0.0688	0.4913	0.074*	
H2B	0.2970	0.0287	0.4722	0.074*	
C3	0.17463 (17)	−0.1307 (2)	0.46633 (6)	0.0656 (4)	
H3A	0.2377	−0.2004	0.4634	0.079*	
H3B	0.1468	−0.1075	0.4332	0.079*	
C4	0.06776 (16)	−0.18753 (18)	0.49661 (6)	0.0584 (4)	
H4A	0.0014	−0.1214	0.4964	0.070*	
H4B	0.0384	−0.2717	0.4813	0.070*	
C5	0.10482 (13)	−0.21730 (13)	0.55016 (6)	0.0475 (3)	
H5A	0.1636	−0.2921	0.5507	0.057*	
H5B	0.0335	−0.2463	0.5688	0.057*	
C6	0.16095 (10)	−0.09053 (12)	0.57496 (5)	0.0365 (2)	
C7	0.20414 (11)	−0.12361 (12)	0.62677 (5)	0.0384 (2)	
C8	0.31007 (12)	−0.10441 (14)	0.65116 (5)	0.0420 (3)	
H8	0.3830	−0.0699	0.6385	0.050*	
C9	0.28579 (12)	−0.14860 (13)	0.70052 (5)	0.0406 (3)	
C10	0.36790 (13)	−0.14279 (14)	0.74392 (5)	0.0440 (3)	
C11	0.46620 (14)	−0.05373 (16)	0.74405 (5)	0.0512 (3)	
H11	0.4817	0.0003	0.7162	0.061*	
C12	0.54184 (16)	−0.04399 (19)	0.78505 (6)	0.0587 (4)	
H12	0.6074	0.0169	0.7844	0.070*	
C13	0.52180 (16)	−0.12314 (19)	0.82699 (6)	0.0603 (4)	
C14	0.42354 (18)	−0.2130 (2)	0.82669 (6)	0.0653 (4)	
H14	0.4087	−0.2677	0.8544	0.078*	
C15	0.34695 (16)	−0.22311 (18)	0.78595 (6)	0.0577 (4)	
H15	0.2812	−0.2838	0.7866	0.069*	
C31	0.6071 (2)	−0.1127 (3)	0.87111 (8)	0.0909 (7)	
H31A	0.6901	−0.1222	0.8601	0.136*	
H31B	0.5885	−0.1845	0.8944	0.136*	
H31C	0.5968	−0.0247	0.8868	0.136*	
O2	0.13845 (8)	0.27957 (9)	0.59456 (4)	0.0446 (2)	
H2	0.0833	0.3217	0.5806	0.067*	
O4	0.37614 (10)	0.53575 (12)	0.59082 (4)	0.0551 (3)	
N2	0.43241 (12)	0.59158 (15)	0.54833 (5)	0.0567 (3)	
C16	0.30531 (13)	0.29329 (15)	0.65160 (6)	0.0474 (3)	
H16A	0.3400	0.2165	0.6334	0.057*	
H16B	0.3721	0.3518	0.6624	0.057*	
C17	0.23731 (17)	0.23905 (17)	0.69701 (6)	0.0587 (4)	
H17A	0.1780	0.1707	0.6865	0.070*	
H17B	0.2949	0.1944	0.7192	0.070*	
C18	0.1719 (2)	0.35391 (19)	0.72487 (6)	0.0650 (4)	
H18A	0.1269	0.3153	0.7526	0.078*	
H18B	0.2316	0.4182	0.7380	0.078*	
C19	0.08429 (16)	0.42955 (17)	0.69051 (6)	0.0570 (4)	
H19A	0.0211	0.3668	0.6793	0.068*	
H19B	0.0453	0.5041	0.7085	0.068*	
C20	0.15192 (13)	0.48696 (13)	0.64607 (5)	0.0439 (3)	
H20A	0.2097	0.5561	0.6573	0.053*	
H20B	0.0939	0.5316	0.6241	0.053*	
C21	0.22078 (11)	0.37544 (12)	0.61712 (5)	0.0383 (2)	
C22	0.29290 (11)	0.44198 (13)	0.57612 (5)	0.0398 (2)	
C23	0.29195 (13)	0.43498 (14)	0.52616 (5)	0.0459 (3)	
H23	0.2431	0.3792	0.5063	0.055*	
C24	0.38164 (12)	0.53111 (14)	0.51042 (5)	0.0437 (3)	
C25	0.41837 (14)	0.56858 (16)	0.45954 (6)	0.0507 (3)	
C26	0.49890 (17)	0.6774 (2)	0.45147 (7)	0.0678 (4)	
H26	0.5283	0.7284	0.4781	0.081*	
C27	0.53507 (19)	0.7092 (3)	0.40341 (8)	0.0829 (6)	
H27	0.5887	0.7822	0.3984	0.099*	
C28	0.4935 (2)	0.6354 (2)	0.36267 (7)	0.0763 (5)	
C29	0.4112 (3)	0.5332 (3)	0.37146 (8)	0.0963 (8)	
H29	0.3792	0.4847	0.3446	0.116*	
C30	0.3732 (3)	0.4987 (2)	0.41912 (7)	0.0801 (6)	
H34	0.3170	0.4280	0.4237	0.096*	
C32	0.5363 (3)	0.6679 (4)	0.31036 (9)	0.1103 (9)	
H32A	0.5810	0.7529	0.3105	0.165*	
H32B	0.4668	0.6761	0.2888	0.165*	
H32C	0.5883	0.5951	0.2987	0.165*	

### Atomic displacement parameters (Å^2^)

**Table:**

	*U*^11^	*U*^22^	*U*^33^	*U*^12^	*U*^13^	*U*^23^
O1	0.0360 (4)	0.0406 (4)	0.0675 (6)	0.0067 (3)	−0.0034 (4)	−0.0048 (4)
O3	0.0416 (5)	0.0563 (5)	0.0471 (5)	−0.0072 (4)	0.0036 (4)	−0.0019 (4)
N1	0.0518 (7)	0.0578 (7)	0.0413 (6)	−0.0064 (5)	0.0042 (5)	0.0006 (5)
C1	0.0346 (6)	0.0643 (8)	0.0443 (7)	−0.0050 (5)	−0.0031 (5)	0.0053 (6)
C2	0.0513 (8)	0.0882 (11)	0.0463 (7)	−0.0129 (8)	−0.0041 (6)	0.0164 (8)
C3	0.0653 (10)	0.0874 (12)	0.0440 (7)	0.0045 (9)	−0.0063 (7)	−0.0033 (8)
C4	0.0627 (9)	0.0580 (8)	0.0546 (8)	−0.0075 (7)	−0.0155 (7)	−0.0070 (7)
C5	0.0533 (7)	0.0382 (6)	0.0510 (7)	−0.0019 (5)	−0.0065 (6)	−0.0037 (6)
C6	0.0306 (5)	0.0353 (5)	0.0436 (6)	0.0027 (4)	−0.0025 (4)	−0.0008 (4)
C7	0.0355 (6)	0.0366 (5)	0.0432 (6)	0.0025 (4)	0.0045 (4)	−0.0038 (5)
C8	0.0369 (6)	0.0478 (6)	0.0413 (6)	0.0019 (5)	0.0017 (5)	0.0031 (5)
C9	0.0441 (6)	0.0372 (6)	0.0404 (6)	0.0040 (5)	0.0039 (5)	−0.0018 (5)
C10	0.0487 (7)	0.0457 (6)	0.0376 (6)	0.0065 (5)	0.0020 (5)	−0.0009 (5)
C11	0.0560 (8)	0.0534 (7)	0.0442 (7)	−0.0014 (6)	−0.0032 (6)	0.0061 (6)
C12	0.0553 (9)	0.0671 (9)	0.0538 (8)	−0.0039 (7)	−0.0081 (6)	0.0015 (7)
C13	0.0632 (9)	0.0777 (11)	0.0400 (7)	0.0091 (8)	−0.0050 (6)	−0.0035 (7)
C14	0.0756 (11)	0.0816 (12)	0.0387 (7)	−0.0003 (9)	0.0005 (7)	0.0116 (7)
C15	0.0626 (9)	0.0641 (9)	0.0465 (7)	−0.0053 (7)	0.0029 (6)	0.0076 (7)
C31	0.0888 (15)	0.132 (2)	0.0516 (10)	−0.0045 (14)	−0.0215 (10)	0.0034 (12)
O2	0.0412 (5)	0.0333 (4)	0.0595 (6)	−0.0003 (3)	−0.0095 (4)	−0.0018 (4)
O4	0.0527 (6)	0.0675 (7)	0.0449 (5)	−0.0229 (5)	−0.0046 (4)	0.0012 (5)
N2	0.0524 (7)	0.0693 (8)	0.0483 (6)	−0.0200 (6)	−0.0015 (5)	0.0057 (6)
C16	0.0452 (7)	0.0445 (6)	0.0523 (7)	0.0060 (5)	−0.0096 (5)	0.0034 (6)
C17	0.0703 (10)	0.0523 (8)	0.0535 (8)	0.0080 (7)	−0.0056 (7)	0.0127 (6)
C18	0.0870 (12)	0.0643 (10)	0.0439 (7)	0.0032 (9)	0.0041 (7)	0.0064 (7)
C19	0.0615 (9)	0.0545 (8)	0.0550 (8)	0.0079 (7)	0.0128 (7)	0.0000 (6)
C20	0.0471 (7)	0.0360 (6)	0.0486 (7)	0.0035 (5)	0.0000 (5)	−0.0009 (5)
C21	0.0363 (6)	0.0338 (5)	0.0446 (6)	0.0007 (4)	−0.0034 (5)	0.0002 (5)
C22	0.0361 (6)	0.0371 (5)	0.0461 (6)	0.0018 (4)	−0.0040 (5)	−0.0010 (5)
C23	0.0481 (7)	0.0426 (6)	0.0470 (7)	−0.0042 (5)	−0.0044 (5)	−0.0050 (5)
C24	0.0403 (6)	0.0431 (6)	0.0476 (7)	0.0030 (5)	0.0005 (5)	−0.0008 (5)
C25	0.0502 (7)	0.0519 (7)	0.0501 (7)	0.0068 (6)	0.0036 (6)	0.0036 (6)
C26	0.0579 (9)	0.0868 (12)	0.0586 (9)	−0.0137 (8)	0.0026 (7)	0.0075 (8)
C27	0.0667 (11)	0.1048 (16)	0.0772 (14)	−0.0099 (10)	0.0099 (10)	0.0285 (12)
C28	0.0845 (13)	0.0901 (14)	0.0544 (9)	0.0206 (11)	0.0148 (9)	0.0135 (10)
C29	0.149 (2)	0.0882 (16)	0.0514 (10)	−0.0098 (16)	0.0070 (13)	−0.0056 (10)
C30	0.1169 (17)	0.0688 (11)	0.0545 (9)	−0.0194 (11)	0.0065 (10)	−0.0061 (9)
C32	0.126 (2)	0.141 (2)	0.0640 (13)	0.0189 (19)	0.0251 (13)	0.0296 (14)

### Geometric parameters (Å, °)

**Table:**

O1—C6	1.4305 (14)	O2—C21	1.4308 (15)
O1—H1	0.8200	O2—H2	0.8200
O3—C7	1.3546 (15)	O4—C22	1.3473 (16)
O3—N1	1.4051 (16)	O4—N2	1.4076 (17)
N1—C9	1.3103 (18)	N2—C24	1.3021 (19)
C1—C6	1.5223 (18)	C16—C17	1.525 (2)
C1—C2	1.526 (2)	C16—C21	1.5342 (17)
C1—H1A	0.9700	C16—H16A	0.9700
C1—H1B	0.9700	C16—H16B	0.9700
C2—C3	1.515 (3)	C17—C18	1.523 (2)
C2—H2A	0.9700	C17—H17A	0.9700
C2—H2B	0.9700	C17—H17B	0.9700
C3—C4	1.528 (3)	C18—C19	1.521 (3)
C3—H3A	0.9700	C18—H18A	0.9700
C3—H3B	0.9700	C18—H18B	0.9700
C4—C5	1.525 (2)	C19—C20	1.513 (2)
C4—H4A	0.9700	C19—H19A	0.9700
C4—H4B	0.9700	C19—H19B	0.9700
C5—C6	1.5294 (17)	C20—C21	1.5325 (18)
C5—H5A	0.9700	C20—H20A	0.9700
C5—H5B	0.9700	C20—H20B	0.9700
C6—C7	1.5072 (17)	C21—C22	1.5027 (18)
C7—C8	1.3450 (18)	C22—C23	1.3469 (19)
C8—C9	1.4215 (17)	C23—C24	1.419 (2)
C8—H8	0.9300	C23—H23	0.9300
C9—C10	1.4747 (19)	C24—C25	1.473 (2)
C10—C11	1.380 (2)	C25—C30	1.375 (3)
C10—C15	1.393 (2)	C25—C26	1.393 (2)
C11—C12	1.383 (2)	C26—C27	1.388 (3)
C11—H11	0.9300	C26—H26	0.9300
C12—C13	1.384 (2)	C27—C28	1.387 (3)
C12—H12	0.9300	C27—H27	0.9300
C13—C14	1.385 (3)	C28—C29	1.362 (4)
C13—C31	1.514 (2)	C28—C32	1.517 (3)
C14—C15	1.384 (2)	C29—C30	1.390 (3)
C14—H14	0.9300	C29—H29	0.9300
C15—H15	0.9300	C30—H34	0.9300
C31—H31A	0.9600	C32—H32A	0.9600
C31—H31B	0.9600	C32—H32B	0.9600
C31—H31C	0.9600	C32—H32C	0.9600
			
C6—O1—H1	109.5	C21—O2—H2	109.5
C7—O3—N1	108.77 (10)	C22—O4—N2	108.51 (11)
C9—N1—O3	105.48 (10)	C24—N2—O4	106.05 (11)
C6—C1—C2	111.35 (11)	C17—C16—C21	111.76 (12)
C6—C1—H1A	109.4	C17—C16—H16A	109.3
C2—C1—H1A	109.4	C21—C16—H16A	109.3
C6—C1—H1B	109.4	C17—C16—H16B	109.3
C2—C1—H1B	109.4	C21—C16—H16B	109.3
H1A—C1—H1B	108.0	H16A—C16—H16B	107.9
C3—C2—C1	111.08 (14)	C18—C17—C16	111.78 (13)
C3—C2—H2A	109.4	C18—C17—H17A	109.3
C1—C2—H2A	109.4	C16—C17—H17A	109.3
C3—C2—H2B	109.4	C18—C17—H17B	109.3
C1—C2—H2B	109.4	C16—C17—H17B	109.3
H2A—C2—H2B	108.0	H17A—C17—H17B	107.9
C2—C3—C4	110.49 (14)	C19—C18—C17	110.50 (14)
C2—C3—H3A	109.6	C19—C18—H18A	109.5
C4—C3—H3A	109.6	C17—C18—H18A	109.5
C2—C3—H3B	109.6	C19—C18—H18B	109.5
C4—C3—H3B	109.6	C17—C18—H18B	109.5
H3A—C3—H3B	108.1	H18A—C18—H18B	108.1
C5—C4—C3	111.66 (13)	C20—C19—C18	110.53 (14)
C5—C4—H4A	109.3	C20—C19—H19A	109.5
C3—C4—H4A	109.3	C18—C19—H19A	109.5
C5—C4—H4B	109.3	C20—C19—H19B	109.5
C3—C4—H4B	109.3	C18—C19—H19B	109.5
H4A—C4—H4B	107.9	H19A—C19—H19B	108.1
C4—C5—C6	111.51 (12)	C19—C20—C21	112.46 (11)
C4—C5—H5A	109.3	C19—C20—H20A	109.1
C6—C5—H5A	109.3	C21—C20—H20A	109.1
C4—C5—H5B	109.3	C19—C20—H20B	109.1
C6—C5—H5B	109.3	C21—C20—H20B	109.1
H5A—C5—H5B	108.0	H20A—C20—H20B	107.8
O1—C6—C7	107.77 (10)	O2—C21—C22	107.39 (10)
O1—C6—C1	111.25 (10)	O2—C21—C20	111.49 (10)
C7—C6—C1	109.89 (10)	C22—C21—C20	109.12 (10)
O1—C6—C5	106.03 (10)	O2—C21—C16	107.32 (10)
C7—C6—C5	110.98 (10)	C22—C21—C16	110.59 (10)
C1—C6—C5	110.82 (11)	C20—C21—C16	110.87 (11)
C8—C7—O3	109.59 (11)	C23—C22—O4	109.43 (12)
C8—C7—C6	133.82 (11)	C23—C22—C21	135.04 (12)
O3—C7—C6	116.46 (10)	O4—C22—C21	115.47 (11)
C7—C8—C9	104.69 (11)	C22—C23—C24	105.05 (12)
C7—C8—H8	127.7	C22—C23—H23	127.5
C9—C8—H8	127.7	C24—C23—H23	127.5
N1—C9—C8	111.47 (12)	N2—C24—C23	110.96 (13)
N1—C9—C10	120.48 (12)	N2—C24—C25	120.07 (13)
C8—C9—C10	127.96 (12)	C23—C24—C25	128.96 (13)
C11—C10—C15	118.49 (13)	C30—C25—C26	118.61 (16)
C11—C10—C9	120.05 (12)	C30—C25—C24	121.05 (15)
C15—C10—C9	121.44 (13)	C26—C25—C24	120.34 (15)
C10—C11—C12	120.75 (14)	C27—C26—C25	119.66 (19)
C10—C11—H11	119.6	C27—C26—H26	120.2
C12—C11—H11	119.6	C25—C26—H26	120.2
C11—C12—C13	121.24 (16)	C28—C27—C26	121.9 (2)
C11—C12—H12	119.4	C28—C27—H27	119.0
C13—C12—H12	119.4	C26—C27—H27	119.0
C12—C13—C14	117.94 (14)	C29—C28—C27	117.17 (18)
C12—C13—C31	120.35 (18)	C29—C28—C32	121.1 (2)
C14—C13—C31	121.69 (17)	C27—C28—C32	121.7 (2)
C15—C14—C13	121.28 (15)	C28—C29—C30	122.3 (2)
C15—C14—H14	119.4	C28—C29—H29	118.9
C13—C14—H14	119.4	C30—C29—H29	118.9
C14—C15—C10	120.30 (16)	C25—C30—C29	120.3 (2)
C14—C15—H15	119.9	C25—C30—H34	119.9
C10—C15—H15	119.9	C29—C30—H34	119.9
C13—C31—H31A	109.5	C28—C32—H32A	109.5
C13—C31—H31B	109.5	C28—C32—H32B	109.5
H31A—C31—H31B	109.5	H32A—C32—H32B	109.5
C13—C31—H31C	109.5	C28—C32—H32C	109.5
H31A—C31—H31C	109.5	H32A—C32—H32C	109.5
H31B—C31—H31C	109.5	H32B—C32—H32C	109.5

### Hydrogen-bond geometry (Å, °)

**Table:**

*D*—H···*A*	*D*—H	H···*A*	*D*···*A*	*D*—H···*A*
O1—H1···O2	0.82	1.98	2.7892 (12)	168
O2—H2···N2^i^	0.82	2.05	2.8629 (16)	173

Symmetry codes: (i) *x*−1/2, −*y*+1, *z*.
